# Exercise Protects against Diet-Induced Insulin Resistance through Downregulation of Protein Kinase Cβ in Mice

**DOI:** 10.1371/journal.pone.0081364

**Published:** 2013-12-09

**Authors:** Xiaoquan Rao, Jixin Zhong, Xiaohua Xu, Brianna Jordan, Santosh Maurya, Zachary Braunstein, Tse-Yao Wang, Wei Huang, Sudha Aggarwal, Muthu Periasamy, Sanjay Rajagopalan, Kamal Mehta, Qinghua Sun

**Affiliations:** 1 College of Public Health, The Ohio State University, Columbus, Ohio, United States of America; 2 Davis Heart & Lung Research Institute, The Ohio State University, Columbus, Ohio, United States of America; 3 Department of Physiology and Cell Biology, The Ohio State University, Columbus, Ohio, United States of America; 4 Department of Molecular and Cellular Biochemistry, The Ohio State University, Columbus, Ohio, United States of America; INSERM/UMR 1048, France

## Abstract

Physical exercise is an important and effective therapy for diabetes. However, its underlying mechanism is not fully understood. Protein kinase Cβ (PKCβ) has been suggested to be involved in the pathogenesis of obesity and insulin resistance, but the role of PKCβ in exercise-induced improvements in insulin resistance is completely unknown. In this study, we evaluated the involvement of PKCβ in exercise-attenuated insulin resistance in high-fat diet (HFD)-fed mice. PKCβ^-/-^ and wild-type mice were fed a HFD with or without exercise training. PKC protein expression, body and tissue weight change, glucose and insulin tolerance, metabolic rate, mitochondria size and number, adipose inflammation, and AKT activation were determined to evaluate insulin sensitivity and metabolic changes after intervention. PKCβ expression decreased in both skeletal muscle and liver tissue after exercise. Exercise and PKCβ deficiency can alleviate HFD-induced insulin resistance, as evidenced by improved insulin tolerance. In addition, fat accumulation and mitochondrial dysfunction induced by HFD were also ameliorated by both exercise and PKCβ deficiency. On the other hand, exercise had little effect on PKCβ^-/-^ mice. Further, our data indicated improved activation of AKT, the downstream signal molecule of insulin, in skeletal muscle and liver of exercised mice, whereas PKCβ deficiency blunted the difference between sedentary and exercised mice. These results suggest that downregulation of PKCβ contributes to exercise-induced improvement of insulin resistance in HFD-fed mice.

## Introduction

Diabetes mellitus, especially type 2 diabetes, is one of the most common chronic diseases worldwide [1]. Diabetes is growing worldwide both in number and significance, due to an increase in economic development and urbanization. Diabetes was reported to affect 366 million people globally in 2011, and this number is expected to rise to 552 million by 2030 in both developed and developing countries [2].

Protein kinase C (PKC) is a family of protein kinases that phosphorylates other proteins at serine and threonine residues [3], [4]. PKC family proteins are involved in multiple cellular processes, including metabolism, differentiation, and cell growth. They are classified into subfamilies, including conventional isoforms (α, β, and γ) that are dependent on both Ca^2+^ and diacylglycerol (DAG) for stimulation, novel isoforms (δ, ε, θ, and η) that are dependent on DAG only, and atypical isoforms (ζ and ι/λ) that are independent of Ca^2+^ and DAG [3], [4]. Abnormal expression of PKC family proteins has been observed in skeletal muscles of patients and animals with diabetes [5], [6], [7]. Among these PKC isoforms, PKCβ protein content was significantly higher, whereas PKCθ and PKCη were significantly lower, in muscle of obese patients compared with muscle of lean control subjects, without a corresponding change in membrane-associated PKC activity [5]. The PKCβ isoform inhibitor ruboxistaurin, which is the most studied PKC inhibitor, has shown some positive effects on diabetes and diabetic complications in clinical trials [8], [9], [10]. Our previous studies demonstrated that PKCβ deficiency alleviated insulin resistance and obesity in mice [11], [12]. Although PKCβ is important in both obesity and insulin resistance, its role in exercise-related changes in HFD-induced metabolic disorders has not yet been reported.

Numerous studies have shown that a high-fat diet (HFD) and sedentary behavior increase the risk of obesity and insulin resistance, whereas increased physical activity reduces this risk [13], [14], [15], [16]. The potential mechanism by which exercise attenuates HFD-induced insulin resistance involves increasing insulin sensitivity and glucose transport into contracting skeletal muscles [17]. However, the underlying molecular mechanisms are not fully understood due to the complicated processes involved in exercise [17]. Given the important regulatory role of PKCβ in insulin resistance, we postulated that it might also play a role in exercise-induced improvement of insulin resistance. We thereby used PKCβ knockout mice and a diet-induced obesity model to test this hypothesis. To our knowledge, this is the first study demonstrating the role of PKCβ in exercise-attenuated insulin resistance by using PKCβ deficiency mice.

## Methods

### Animals and diet

Production of PKCβ^-/-^ mice in C57BL/6J background and genotypic determination were performed as described previously [18]. At the age of four weeks, PKCβ^-/-^ and wild-type (WT) C57BL/6J mice were fed a high-fat diet (HFD) containing 42% of calories from fat (TD88137, Harlan, Madison, WI). At the age of 12 weeks, both WT and PKCβ^-/-^ mice were randomly assigned into sedentary (SED) or exercise (EX) group for 8 weeks (Figure S1). All mice were allowed to eat and drink *ad libitum* throughout the duration of the study. The mice were housed on a 12∶12-hour light-dark cycle in a temperature and humidity controlled vivarium. National Institutes of Health guidelines for the care and use of laboratory animals were strictly followed, and all experiments were approved by the Animal Care and Use Committee at The Ohio State University.

### Exercise intervention

Exercise intervention was performed as described previously [19]. Briefly, mice in exercise group were exercise-trained on a motorized treadmill (Columbus Instruments, Columbus, OH) at a speed of 15 m/min, 40 min/day, and 5 days/week for 8 weeks. Mice in SED group were put on the same treadmill without running for 40 min/day and 5 days/week for 8 weeks.

### Body weight, tissue weight, food intake and water intake

Body weight, food intake, and water intake were recorded weekly during the exercise intervention. 24 hours after the end of the exercise intervention, all mice were fasted overnight and euthanized by CO_2_ inhalation overdose. Blood samples were obtained and plasma was collected and stored at −80°C immediately. Heart, liver, calf muscles (gastrocnemius and soleus), thigh muscle (quadriceps femoris and adductor magnus), epididymal fat, inguinal fat, together with brown adipose tissue from the interscapular depot were carefully excised. All the tissue samples were weighed and then immediately frozen in liquid nitrogen.

### Magnetic resonance imaging (MRI)

Body fat mass (abdominal cavity) was evaluated by *in vivo* MRI, as described previously [19]. Briefly, 11.7 T small bore vertical NMR system (BioSpec, Bruker, Ettlingen, Germany) was used. First, mouse was anesthetized with isoflurane (1.5–2.0%) and placed in a 30-mm birdcage coil. After the mouse was positioned in the scanner, a coronal spin-echo localizing sequence was used to identify both kidneys. Finally, from the superior pole of the uppermost kidney to the caudal aspect of the mouse, thirty contiguous 1-mm thick axial slices were obtained using a spin-echo sequence with a 256×256 pixel size (30×30 mm). Data were analyzed by National Institutes of Health Image J software.

### Glucose tolerance test (GTT) and insulin tolerance test (ITT)

After 8 weeks exercise intervention, a glucose tolerance test (overnight fasting) and insulin tolerance test (6 hours fasting) were performed on all mice as previously described [20]. Briefly, mice were weighed and then injected intraperitoneally with either glucose (2 mg/kg body weight) or insulin (0.5 U/kg body weight). Blood samples were collected through the tail vein and glucose concentrations were measured before and 30, 60, 90 and 120 min after the challenge on an Elite Glucometer (Bayer, Leverkusen, Germany). Area under the curve was calculated using GraphPad software.

### Plasma insulin, leptin, adiponectin level and insulin resistance assessment

After overnight fasting, blood samples were collected into EDTA-coated tubes and plasma was collected after centrifugation at 2000×g for 15 min. Plasma insulin level was determined following a standard protocol of an ultrasensitive Mouse Insulin ELISA kit (Crystal Chem, Downers Grove, IL) [21]. Leptin level was determined following a standard protocol of the Quantikine Mouse Leptin ELISA kit (R&D, Minneapolis, MN). Adiponectin level was measured according to the manufacturer's instructions using the Quantikine Mouse Adiponectin/Acrp30 ELISA kit (R&D, Minneapolis, MN). Insulin resistance (IR) was calculated using the homeostasis model assessment (HOMA) method based on the formula 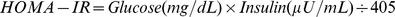 [22].

### Oxygen consumption and CO_2_ production measurements

Oxygen consumption and CO_2_ production were measured simultaneously for each mouse using a computer-controlled, Comprehensive Lab Animal Monitoring (CLAMS) System (Columbus Instruments, Columbus, OH) [23]. Each mouse was measured individually in a resting state for 24 hours at 22°C in presence of food and water or measured individually when running on a treadmill at a speed of 15 m/min for 40 min.

### Measurement of blood inflammatory biomarkers

At the end of the study, blood was collected and plasma was stored at −80°C for the analysis of cytokines. Plasma levels of TNF-α, IFN-γ, and monocyte chemoattractant protein 1 (MCP-1) were measured using Mouse Inflammation 6-Plex Kit from BD Bioscience (San Diego, CA), according to manufacturer's instructions. The cytokine levels were then determined using a BD LSR II instrument and analyzed by the BD CBA software (BD Biosciences, San Jose, CA) [21].

### Transmission electron microscopy

To investigate the mitochondrial changes *in situ* between groups, we examined the ultrastructure of mitochondria by transmission electron microscopy (TEM) as previously described [19]. Briefly, muscle tissue was excised into small pieces (around 1 mm^3^) and fixed in 2.5% gluteraldehyde (0.1 M phosphate buffer, pH 7.4) for 3 hours. Then each specimen was post-fixed in 1% osmium tetroxide for 1 hour and dehydrated through a graded ethanol series (50–100%). After embedded in eponate 12 resin, sections at a thickness of 80 nm were cut and stained by 2% aqueous uranyl acetate followed by lead citrate. The grids were loaded and observed in a Tecnai G2 Spirit transmission electron microscope (FEI, Hillsboro, OR). The images of mitochondria were captured at a magnification of 18,500×. For the morphometric analysis, five micrographs per tissue were counted. Mitochondrial size and number were analyzed by National Institutes of Health Image J software.

### Immunoblotting

Mice tissues were collected after overnight fasting. Tissue lysates from the soleus muscle and liver were prepared in a lysis buffer containing 50 mM Tris·HCl (pH 8.0), 2 mM EGTA, 1% SDS, and protease inhibitors. After adjusting the protein concentration, the samples were loaded, and proteins were separated by 10% SDS/PAGE gel electrophoresis and transferred to PVDF membrane. The membranes were incubated with specific primary antibodies against PKCα, PKCλ (BD Transduction Laboratories, Lexington, KY), phospho-AKT, AKT (Cell Signaling Technology, Danvers, MA; 1∶1000 dilution), PKCβ, PKCδ, PKCε, β-actin (Santa Cruz, Santa Cruz, CA; 1∶200 dilution), and GAPDH (eBioscience, San Diego, CA; 1∶1000 dilution), followed by visualization using horseradish peroxidase-conjugated specific secondary antibodies. All immunoreactive bands were detected by SuperSignal substrate kit (Thermo Fisher, Rockford, IL).

### Data analyses

All data are expressed as means ± SEM unless otherwise specified. Difference between two groups was tested by student's t test. Differences among groups were tested by two-way ANOVA and Boneferroni's post hoc test using GraphPad Prism ver. 5 (GraphPad Software, La Jolla, CA). *P* values of <0.05 were considered statistically significant.

## Results

### Decreased expression of PKCβ in both skeletal muscle and liver after exercise

**Figure 1:**
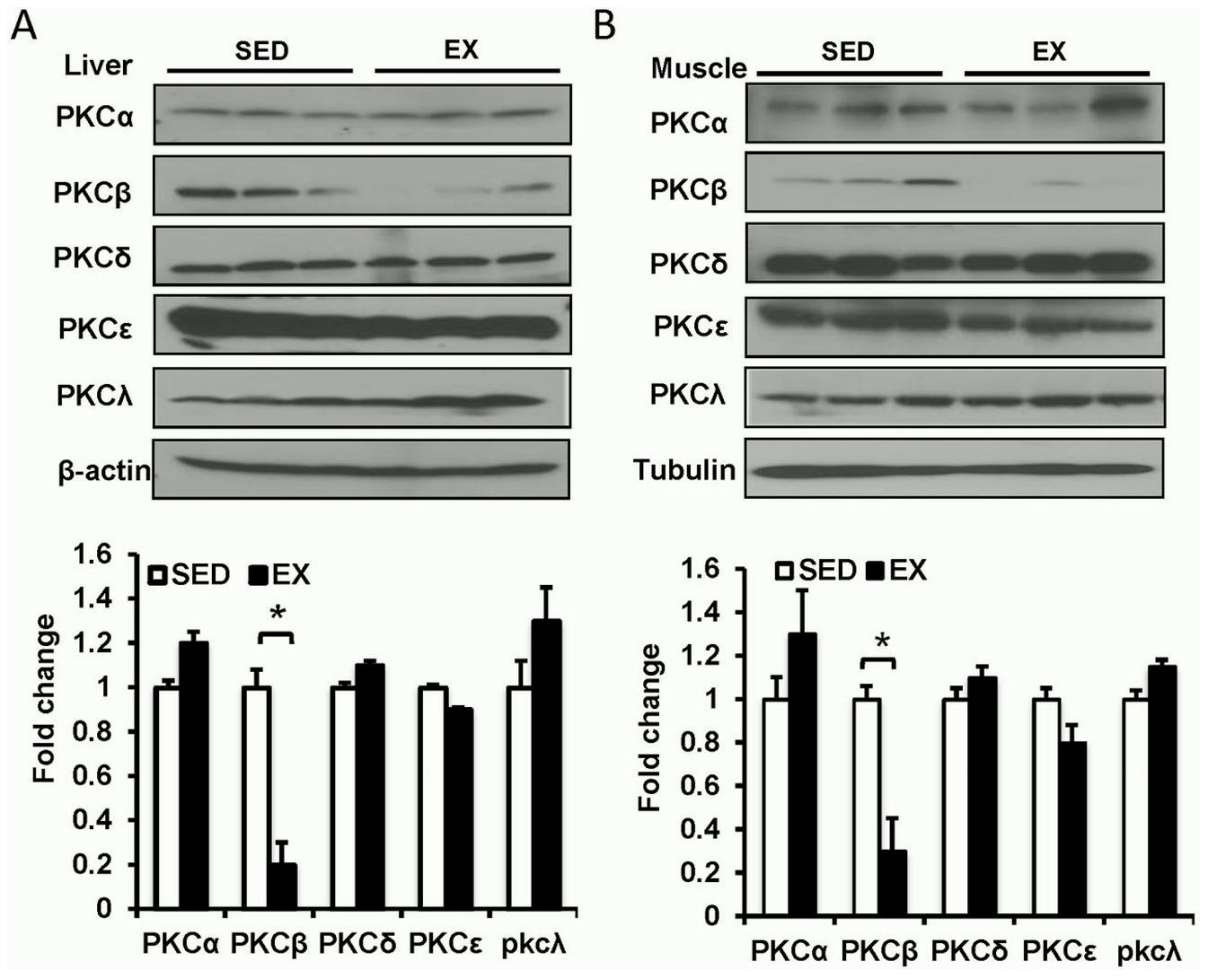
Expression of PKC isoforms in HFD and exercised mice. A, Expressions of PKC isoforms in liver. Wild-type (WT) mice fed on a high-fat diet (HFD) feeding were either exercised (EX) or sedentary (SED) for 8 weeks. Liver was isolated and used for Western blot detection of PKC isoforms (Top panel, representative images; bottom panel, statistical analysis). *, *P*<0.05. B, Expressions of PKC isoforms in skeletal muscle. WT mice fed on a HFD were either exercised or sedentary for 8 weeks. Skeletal muscle tissue was isolated and used for Western blot detection of PKC isoforms (Top panel, representative images; bottom panel, statistical analysis). *, *P*<0.05.

PKC proteins have been suggested to play a role in the insulin sensitivity of skeletal muscle [5], [6], [7]. To investigate the involvement of PKC in exercise, we tested the expression of different isoforms in the skeletal muscle and liver after 8 weeks of exercise. As depicted in Figures 1A & 1B, no significant differences of PKCα or δ between exercise and sedentary groups were detected in both skeletal muscle and liver in WT mice. PKCλ was increased in muscle and liver after exercise. PKCε level was slightly decreased in the muscle but was not changed in the liver of exercised mice compared with sedentary controls. It is noteworthy that PKCβ was significantly reduced in both the skeletal muscle (SED 1±0.08 vs. EX 0.21±0.09, p<0.05) and liver (SED 1±0.06 vs. EX 0.3±0.15, p<0.05) of exercised mice. These data suggested that PKCβ might be involved in exercise-induced metabolic changes.

### Both exercise and PKCβ deficiency ameliorated HFD-induced obesity

**Figure 2:**
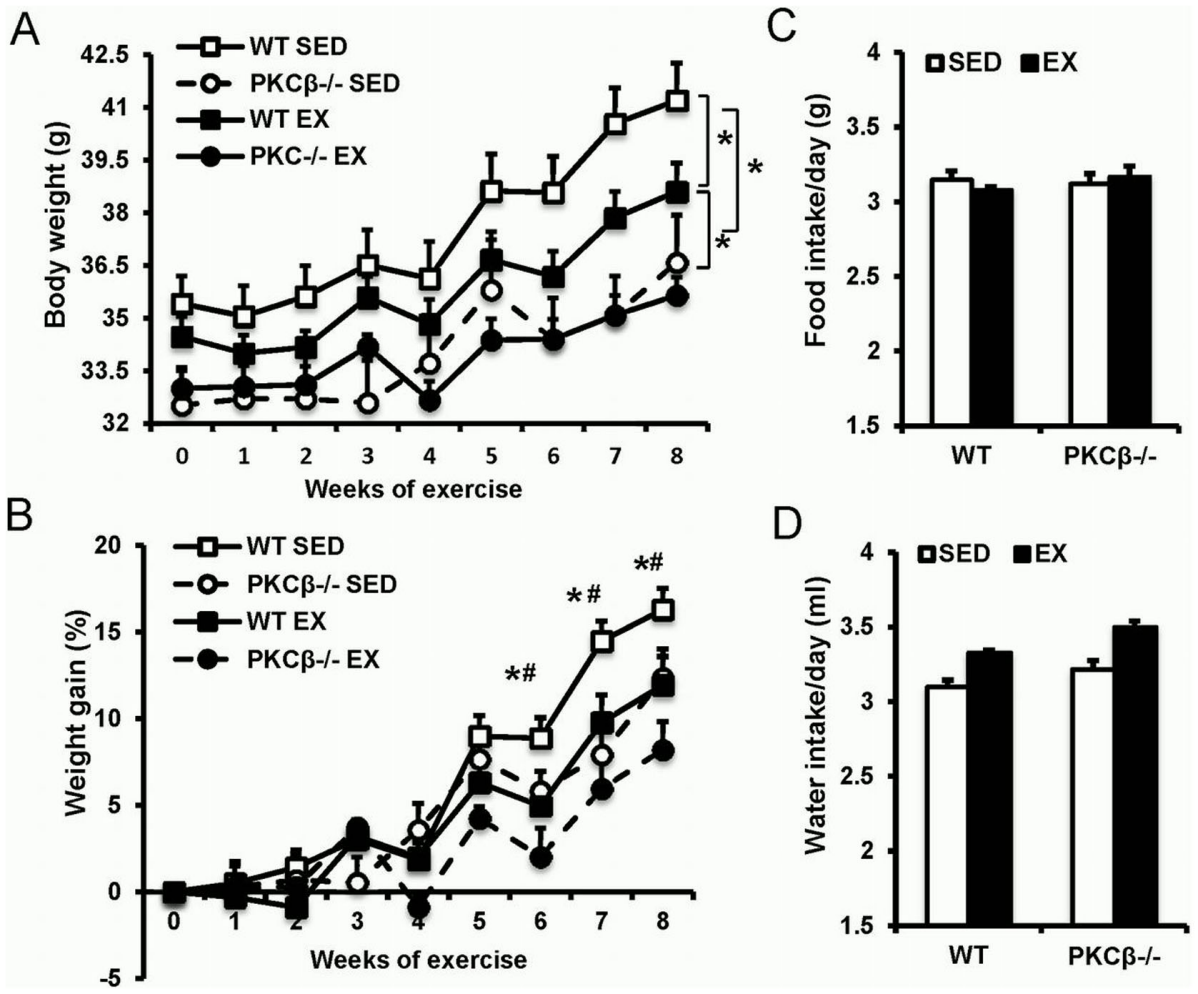
Changes in body weight, food and water intake following HFD and exercise. A, Effect of exercise on body weight of WT and PKCβ^-/-^ mice. Mice were weighted once a week during the exercise-training period. B, Weight gain of WT and PKCβ^-/-^ mice during the exercise-training period. Weight gain was less in WT mice but not in PKCβ^-/-^ mice after exercise. C & D, Food intake (C) and water intake (D) of WT and PKCβ^-/-^ mice with or without exercise. WT, wild-type; EX, exercise; SED, sedentary; Data are shown as mean ± SEM; n = 8 for each group; *, *P*<0.05 WT vs. PKCβ^-/-^; #, *P*<0.05 EX vs. SED.

After eight weeks of exercise, exercised WT mice had a less body weight compared with WT mice (WT SED 41.2±4.2 g vs. WT EX 38.6±3.2 g after 8 weeks of exercise, p<0.05). The body weights of both sedentary and exercised PKCβ^-/-^ mice were lower than that of corresponding WT mice (WT SED 41.2±4.2 g vs. PKCβ^-/-^ SED 36.6±5.4 g, p<0.05; WT EX 38.6±3.2 g vs. PKCβ^-/-^ EX 35.6±2.1 g, p<0.05). Interestingly, we failed to detect a significant difference in body weight between exercise PKCβ^-/-^ and sedentary PKCβ^-/-^ mice (Figure 2A). The total weight gain of sedentary WT mice was significantly higher than in the other groups, while no significant difference of weight gain was observed between exercised WT and exercised PKCβ^-/-^ mice (WT SED 16.3±1.2 g vs. WT EX 11.9±1.7 g vs. PKCβ^-/-^ SED 12.4±1.7 g vs. PKCβ^-/-^ EX 8.2±1.6 g, p<0.05; Figure 2B). Despite lower body weight, the daily food and water intake of PKCβ^-/-^ mice was similar to that of WT mice (Food intake: WT SED 3.14±0.06 g vs. WT EX 3.08±0.02 g vs. PKCβ^-/-^ SED 3.12±0.07 g vs. PKCβ^-/-^ EX 3.17±0.07 g, p>0.05; Water intake: WT SED 3.10±0.04 mL vs. WT EX 3.33±0.15 mL vs. PKCβ^-/-^ SED 3.22±0.06 mL vs. PKCβ^-/-^ EX 3.50±0.04 mL, p>0.05; Figures 2C & 2D).

**Figure 3:**
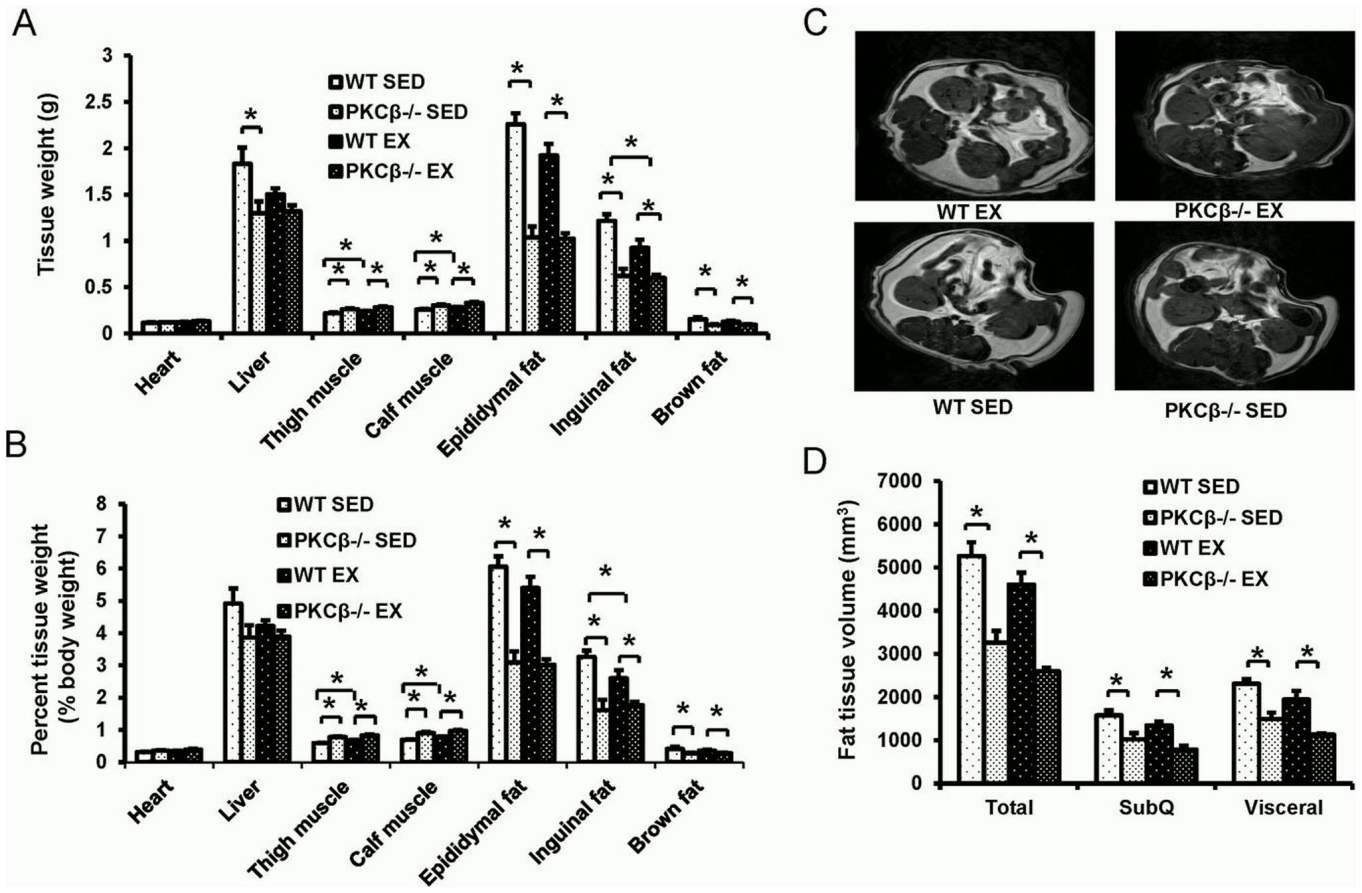
Changes in fat and muscle tissue after exercise in WT and PKCβ^-/-^ mice. A, Tissue weights in WT and PKCβ^-/-^ mice with or without exercise. Muscle (thigh and calf) weights were increased and inguinal fat weight decreased in WT mice after exercise but not in PKCβ^-/-^ mice. PKCβ^-/-^ mice had higher muscle weight and lower fat weight compared to WT mice. B, Percent tissue weight over body weight in WT and PKCβ^-/-^ mice with or without exercise. Exercise increased muscle percentage and decreased percentage of inguinal fat weight in WT mice but not in PKCβ^-/-^ mice. PKCβ^-/-^ mice had higher muscle weight and lower fat weight compared to WT mice with the same intervention. C, Representative images of magnetic resonance imaging (MRI) of abdominal cavity. D, Quantitative analysis of fat volume as determined by MRI. PKCβ^-/-^ mice had significantly lower fat volumes of total, subcutaneous (SubQ), and visceral fats than WT mice and exercise did not significantly change it compared to sedentary (SED) intervention. WT, wild-type; EX, exercise; SED, sedentary; Data are shown as mean ± SEM; N = 8, *, *P*<0.05.

Exercise reduced fat mass and increased skeletal muscle weight in WT mice but not PKCβ^-/-^ mice. Compared to WT controls, PKCβ^-/-^ mice (both exercised and sedentary) had slightly increased tissue weights of skeletal muscles and significantly reduced tissue weights of inguinal fat, epididymal fat, and interscapular brown fat. The liver weight of sedentary WT mice was also higher than that of sedentary PKCβ^-/-^ mice, while no significant difference of liver weight was observed among other groups (Figure 3A). Similar results of tissue weight were obtained when normalized to body weight (Figure 3B). Consistently, MRI scans also suggested that both the visceral and subcutaneous fat masses of PKCβ^-/-^ mice were less than that of WT mice (Figures 3C & 3D)

### Exercise improved insulin resistance via reducing PKCβ

**Figure 4:**
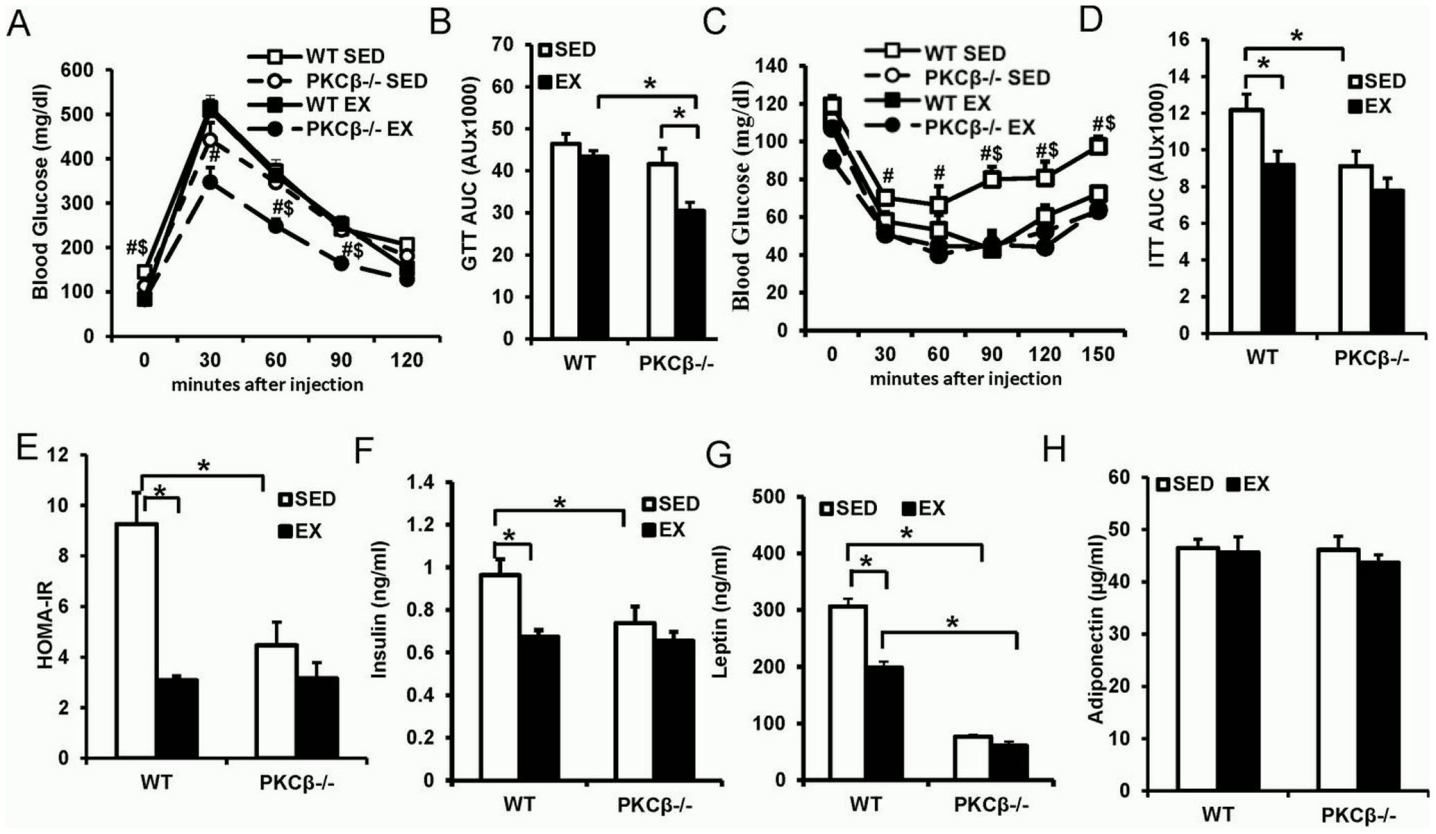
Effect of exercise and PKCβ deficiency on insulin resistance. A & B, Intraperitoneal glucose tolerance test (IPGTT) in WT and PKCβ^-/-^ mice with or without exercise. A, Blood glucose responses; B, Area under the curve (AUC) of IPGTT. C & D, Intraperitoneal insulin tolerance test (IPITT) in WT and PKCβ^-/-^ mice with or without exercise. C, Blood glucose curve; D, Area under the curve (AUC) of IPITT. E, Homeostasis model assessment-estimated insulin resistance (HOMA-IR) in WT and PKCβ^-/-^ mice with or without exercise. HOMA-IR was calculated using the formula HOMA-IR  =  fasting glucose (mg/dl) x fasting insulin (µU/mL)/405. F, Fasting serum insulin level. After 16 h of fasting, serum was collected for ELISA detection of insulin. Exercise decreased fasting insulin level in WT but not in PKCβ^-/-^ mice. Insulin level in sedentary PKCβ^-/-^ mice was lower than that of sedentary WT mice. G, Fasting serum leptin level. After 16 h of fasting, serum was collected for ELISA detection of leptin. Exercise decreased fasting leptin level in WT but not in PKCβ^-/-^ mice. Leptin level in sedentary PKCβ^-/-^ mice was lower than that of sedentary WT mice. H, Fasting serum adiponectin level. After 16 h of fasting, serum was collected for ELISA detection of leptin. No significant difference of adiponectin was detected among the 4 groups. WT, wild-type; EX, exercise; SED, sedentary; Data are expressed as mean ± SEM; n = 8, #, *P*<0.05, WT vs. PKCβ^-/-^; $, *P*<0.05, EX vs. SED; *, *P*<0.05.

Upon glucose challenge, blood glucose levels in exercised PKCβ^-/-^ mice were significantly lower than those of other groups (AUC: WT SED 46.41±2.38 vs. WT EX 43.38±1.42 vs. PKCβ^-/-^ SED 41.62±3.70 vs. PKCβ^-/-^ EX 30.49±2.04, p<0.05; Figures 4A & 4B). Interestingly, both ITT and HOMA-IR results showed the insulin sensitivity in sedentary WT mice was lower than that of other groups while no significant difference was observed among the rest of the groups (ITT AUC: WT SED 12.18±0.86 vs. WT EX 9.19±0.73 vs. PKCβ^-/-^ SED 9.11±0.81 vs. PKCβ^-/-^ EX 7.77±0.68, p<0.05; HOMA-IR: WT SED 9.26±1.87 vs. WT EX 3.09±0.14 vs. PKCβ^-/-^ SED 4.47±1.13 vs. PKCβ^-/-^ EX 3.16±0.92, p<0.05; Figure 4C–E). Plasma insulin and leptin levels of sedentary WT mice were higher than those of rest groups, while no significant difference between exercised and sedentary PKCβ^-/-^ mice was detected (Insulin: WT SED 0.97±0.07 ng/mL vs. WT EX 0.67±0.03 ng/mL vs. PKCβ^-/-^ SED 0.74±0.08 ng/mL vs. PKCβ^-/-^ EX 0.66±0.04 ng/mL, p<0.05; Leptin: WT SED 306.5±9.9 ng/mL vs. WT EX 198.8±13.7 ng/mL vs. PKCβ^-/-^ SED 76.7±7.6 ng/mL vs. PKCβ^-/-^ EX 60.7±3.7 ng/mL, p<0.05; Figures 4F & 4G). Similar plasma adiponectin levels were found among the four groups (Figure 4H). Collectively, these results demonstrated PKCβ deficiency enhances insulin sensitivity with no further improvement by exercise, suggesting exercise-induced improvements in insulin resistance may involve PKCβ-mediated pathways.

### Exercise increased metabolic rate via decrease in PKCβ

**Figure 5:**
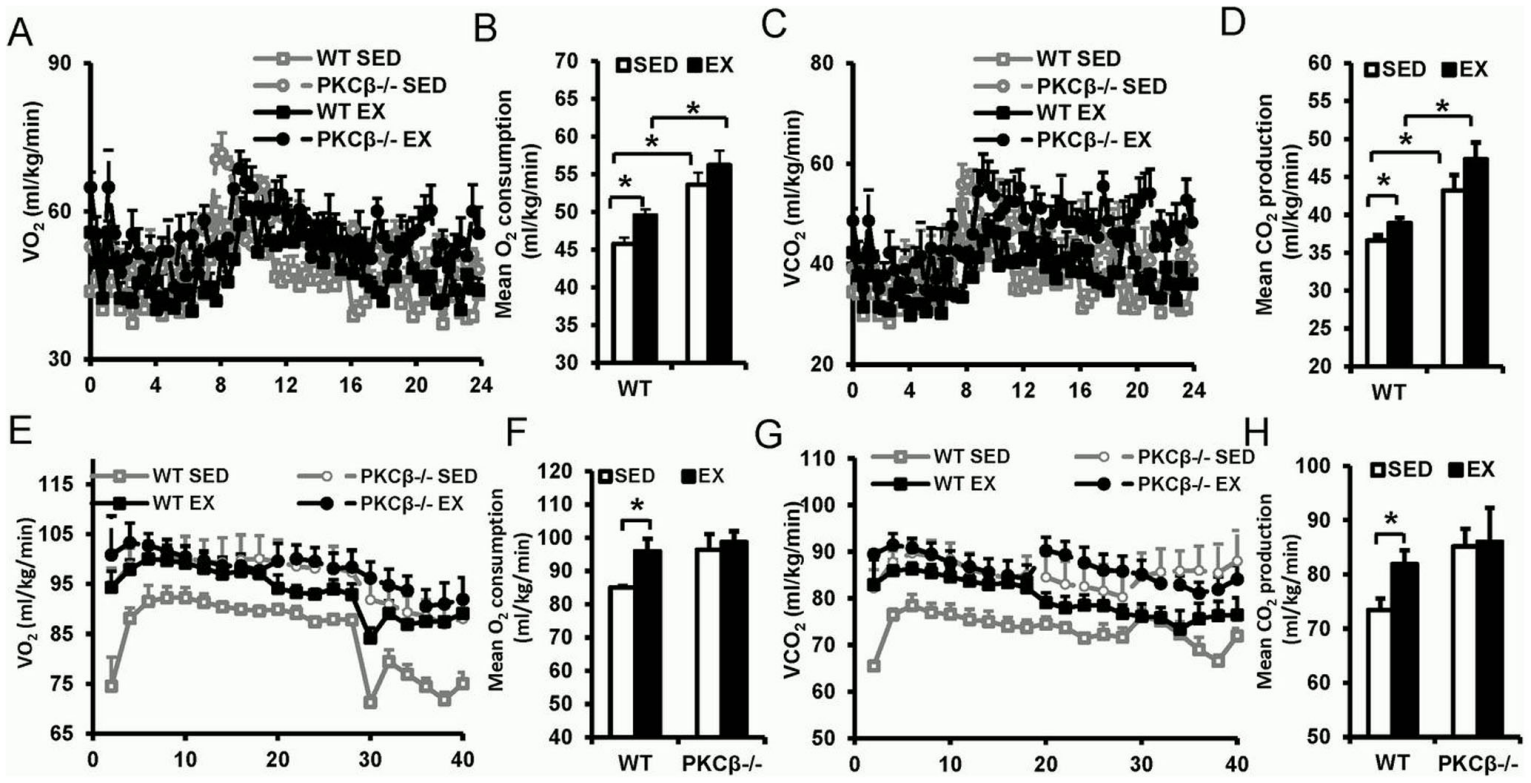
Metabolism increased after exercise in WT but not in PKCβ^-/-^ mice. A, Resting O_2_ consumption over 24 hours. O_2_ consumption of WT and PKCβ^-/-^ mice with or without exercise was measured in a resting state for 24 hours using a computer-controlled, Comprehensive Lab Animal Monitoring System (CLAMS). B, Average resting O_2_ consumption in WT and PKCβ^-/-^ mice with or without exercise. C, Resting CO_2_ production over 24 hours. CO_2_ production of WT and PKCβ^-/-^ mice with or without exercise was measured in a resting state for 24 hours at 22°C in presence of food and water using CLAMS. D, Average resting CO_2_ production in WT and PKCβ^-/-^ mice with or without exercise. E, O_2_ consumption with exercise intervention. O_2_ consumption of WT and PKCβ^-/-^ mice with or without exercise was measured. F, Average exercise O_2_ consumption in WT and PKCβ^-/-^ mice with or without exercise. G, CO_2_ production with exercise intervention. CO_2_ production of WT and PKCβ^-/-^ mice with or without exercise was measured. H, Average exercise CO_2_ production in WT and PKCβ^-/-^ mice with or without exercise. WT, wild-type; EX, exercise; SED, sedentary; Data are expressed as mean ± SEM; n = 5, *, *P*<0.05.

As shown in Figures 5A–D, exercise increased resting O_2_ consumption and CO_2_ production in WT mice, while it had no significant impact on metabolic rate of PKCβ^-/-^ mice. PKCβ^-/-^ mice had a higher O_2_ consumption and CO_2_ production than WT mice, regardless of exercise or sedentary (O_2_ consumption: WT SED 45.77±0.87 mL/kg/min vs. WT EX 49.54±0.81 mL/kg/min vs. PKCβ^-/-^ SED 53.60±1.61 mL/kg/min vs. PKCβ^-/-^ EX 56.22±1.92 mL/kg/min, p<0.05; CO_2_ production: WT SED 36.62±0.67 mL/kg/min vs. WT EX 38.92±0.70 mL/kg/min vs. PKCβ^-/-^ SED 43.23±2.03 mL/kg/min vs PKCβ^-/-^ EX 47.36±2.18 mL/kg/min, p<0.05). Consistently, exercised WT mice had a higher metabolic rate than sedentary WT mice when mice run on treadmill. Comparable O_2_ consumption and CO_2_ production were detected in exercised and sedentary PKCβ^-/-^ mice when mice run on treadmill (O_2_ consumption: WT SED 85.07±0.46 mL/kg/min vs. WT EX 95.97±3.73 mL/kg/min vs. PKCβ^-/-^ SED 96.39±4.74 mL/kg/min vs. PKCβ^-/-^ EX 98.72±3.23 mL/kg/min, p<0.05; CO_2_ production: WT SED 73.43±2.11 mL/kg/min vs. WT EX 81.92±2.49 mL/kg/min vs. PKCβ^-/-^ SED 85.16±3.22 mL/kg/min vs PKCβ^-/-^ EX 86.02±6.22 mL/kg/min, p<0.05; Figures 5E–H). These results indicated that exercise increases metabolic rate possibly through decreasing PKCβ.

### Both exercise and PKCβ deficiency reduced HFD-induced mitochondrial defects in the skeletal muscle

**Figure 6:**
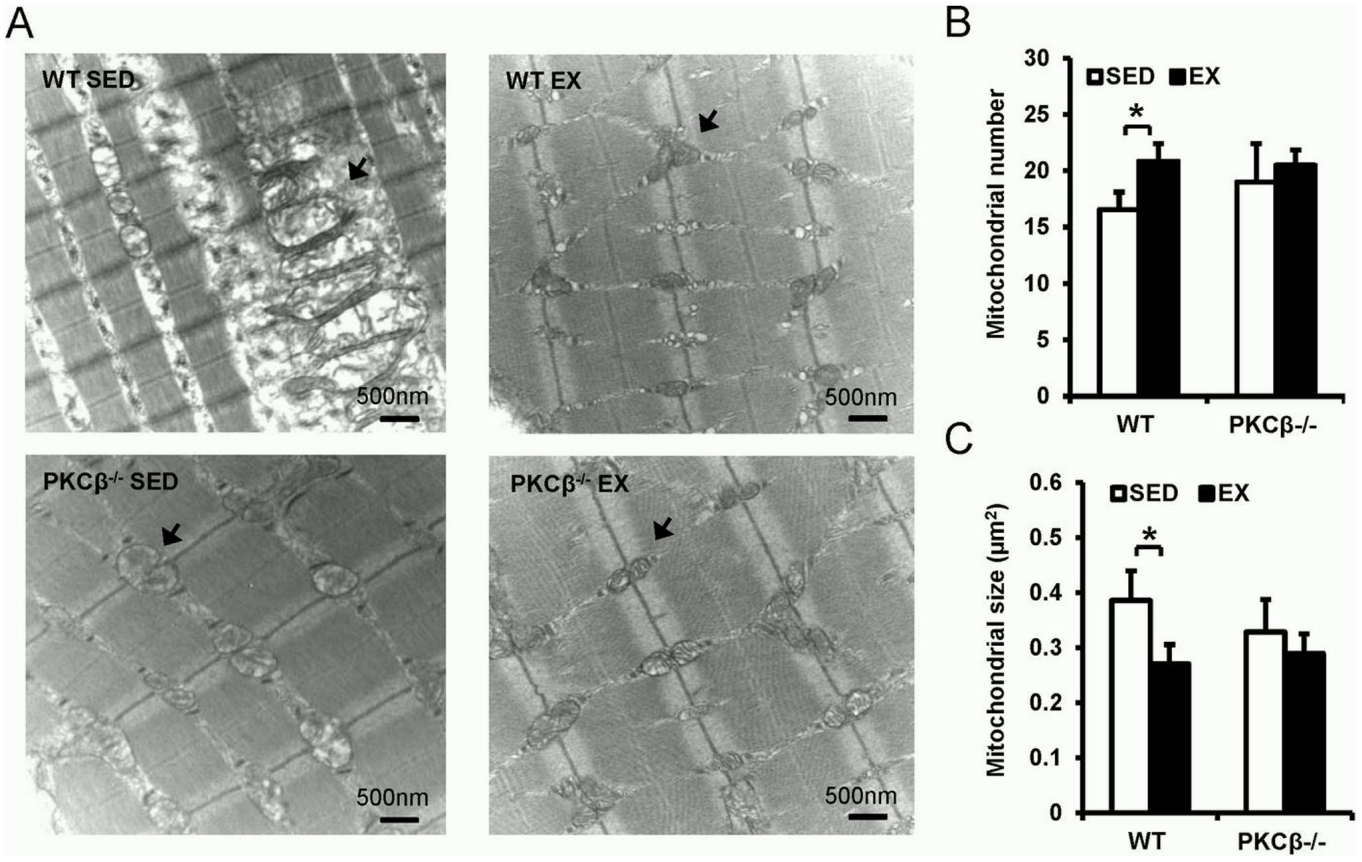
Both exercise and PKCβ deficiency reduced high-fat diet-induced mitochondrial dysfunction in the skeletal muscle. A, Representative images of transmission electronic microscopy (TEM) show mitochondrial abnormality in the skeletal muscle of WT SED mice and reduced mitochondrial damage in the other 3 groups. Note the swollen and decreased matrix density of mitochondrial in WT SED group. Bar: 500 nm; Magnification: 18500×; The arrows indicate mitochondria. B, Mitochondrial number analysis shows that exercise increased mitochondrial number in WT but not in PKCβ^-/-^ mice. Six images per mice, 3 mice per group have been counted and quantified. C, Mitochondrial size analysis shows that mitochondrial size was significantly increased in WT SED group but not in the other 3 groups. Six images per mice, 3 mice per group have been counted and quantified. WT, wild-type; EX, exercise; SED, sedentary; Data are presented as mean ± SEM; *, *P*<0.05.

As shown in Figure 6A, mitochondria from the muscle in exercised WT mice had a more clearly defined internal membrane structure, including wider cristae, than those from sedentary WT mice. Mitochondrial number in the skeletal muscle of exercised WT mice was also increased compared with sedentary WT mice. However, similar mitochondrial numbers were found in exercised and sedentary PKCβ^-/-^ mice (WT SED 16.56±1.55 vs. WT EX 20.85±1.57 vs. PKCβ^-/-^ SED 19.00±3.40 vs. PKCβ^-/-^ EX 20.50±1.33, p<0.05; Figure 6B). Furthermore, the mitochondria from skeletal muscle of sedentary WT mice were found to be enlarged and disordered in comparison to that of exercised WT mice, while exercised and sedentary PKCβ^-/-^ mice had mitochondria with similar size to exercised WT mice (WT SED 0.39±0.05 µm^2^ vs. WT EX 0.27±0.04 µm^2^ vs. PKCβ^-/-^ SED 0.33±0.06 µm^2^ vs. PKCβ^-/-^ EX 0.29±0.03 µm^2^, p<0.05; Figures 6A & 6C).

### Eight weeks of exercise did not have significant impact on adipose tissue inflammation

**Figure 7:**
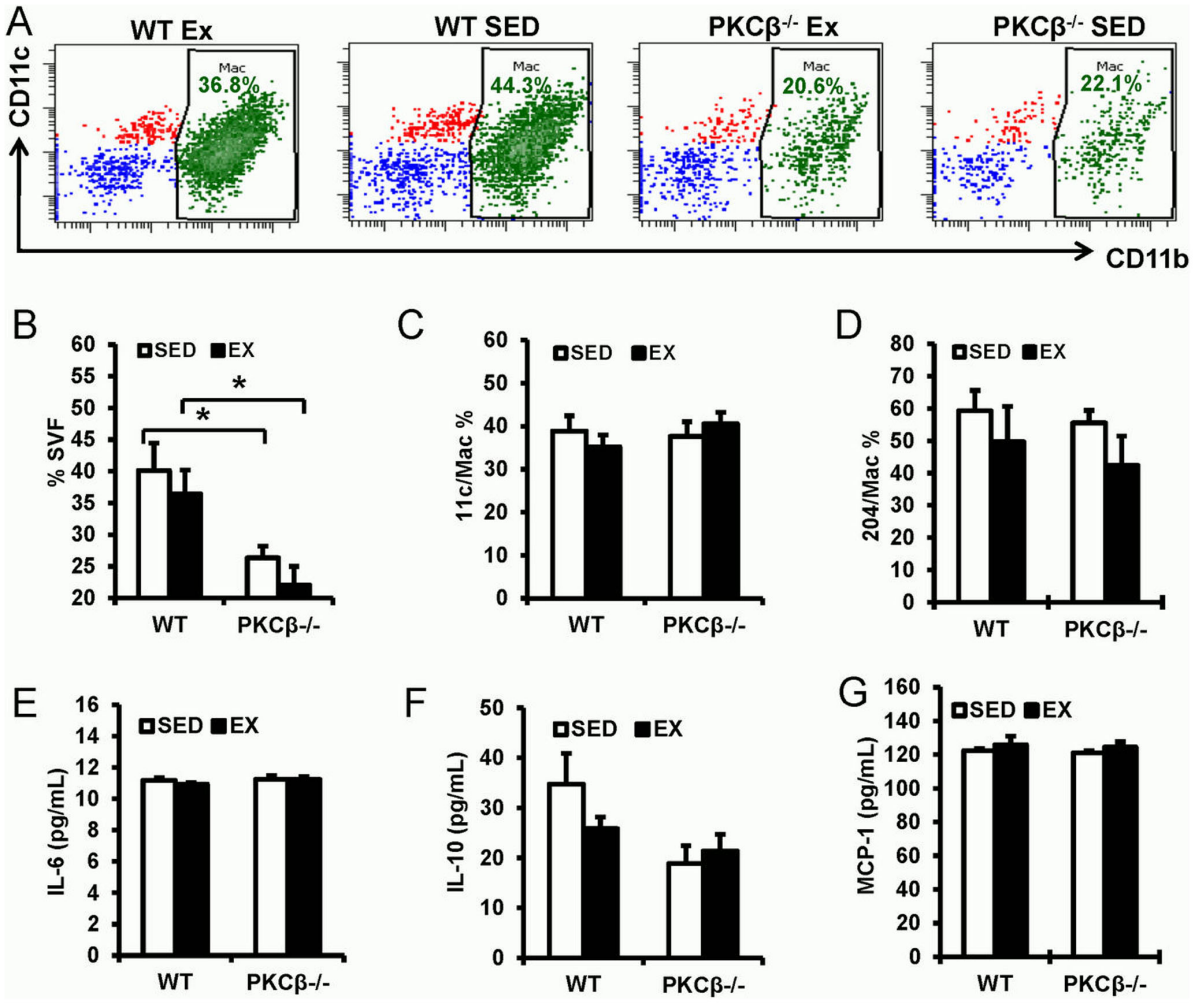
Effect of exercise and PKCβ deficiency on adipose tissue inflammation and plasma cytokines. A–D, Adipose inflammation in WT and PKCβ^-/-^ mice with or without exercise. Exercise slightly reduced the infiltration of macrophages although not statistically significant. Macrophage percentage in SVF was significantly lower in PKCβ^-/-^ mice. Macrophages with M1 or M2 phenotype were not affected by exercise or PKCβ deficiency. A, Representative flow cytometric plots of adipose tissue macrophages; B, flow statistical analyses of adipose tissue macrophages; C, Percentage of classically activated macrophages (M1, CD11b^+^ CD11c^+^) in adipose tissue macrophages; D, Percentage of alternatively activated macrophages (M2, CD11b^+^ CD204^+^) in adipose tissue macrophages. E–G, Plasma cytokine levels in WT and PKCβ^-/-^ mice with or without exercise. Plasma isolated from exercise or sedentary mice was collected for inflammatory cytokine detection using BD™ Cytometric Bead Array Mouse Inflammation Kit. Exercise and PKCβ deficiency do not affect the plasma level of IL-6, IL-10, and MCP-1. E, Plasma IL-6 level; F, Plasma IL-10 Level; G, Plasma MCP-1 Level. WT, wild-type; EX, exercise; SED, sedentary; Data are presented as mean ± SEM; n = 5, *, *P*<0.05.

Increased ratio of M1 (classically activated macrophages) versus M2 (alternatively activated macrophages) is suggested to be an important feature of adipose inflammation and insulin resistance [24]. To investigate the significance of exercise on adipose tissue inflammation, we detected the macrophage percentage and M1/M2 ratio (CD11b^+^ CD11c^+^ cell/CD11b^+^ CD204^+^ cell) in stromal vascular fraction (SVF) of epididymal fat. As shown in Figures 7A & 7B, exercise slightly decreased macrophage numbers in epididymal SVF from WT mice although not to a statistically significant level. Deficiency of PKCβ reduced macrophage infiltration in epididymal fat (WT SED 40.13±4.29% vs. WT EX 36.45±3.75% vs. PKCβ^-/-^ SED 26.36±1.85% vs. PKCβ^-/-^ EX 22.08±2.91%, p<0.05). Mice from all the four groups had similar levels of classical and alternative macrophage activation (Figures 7C & 7D). In addition, the plasma levels of cytokines, including IL-6, IL-10, and MCP-1, were comparable among all the four groups (Figures 7E–G).

### Both exercise and PKCβ deficiency enhanced insulin signaling in peripheral tissues

**Figure 8:**
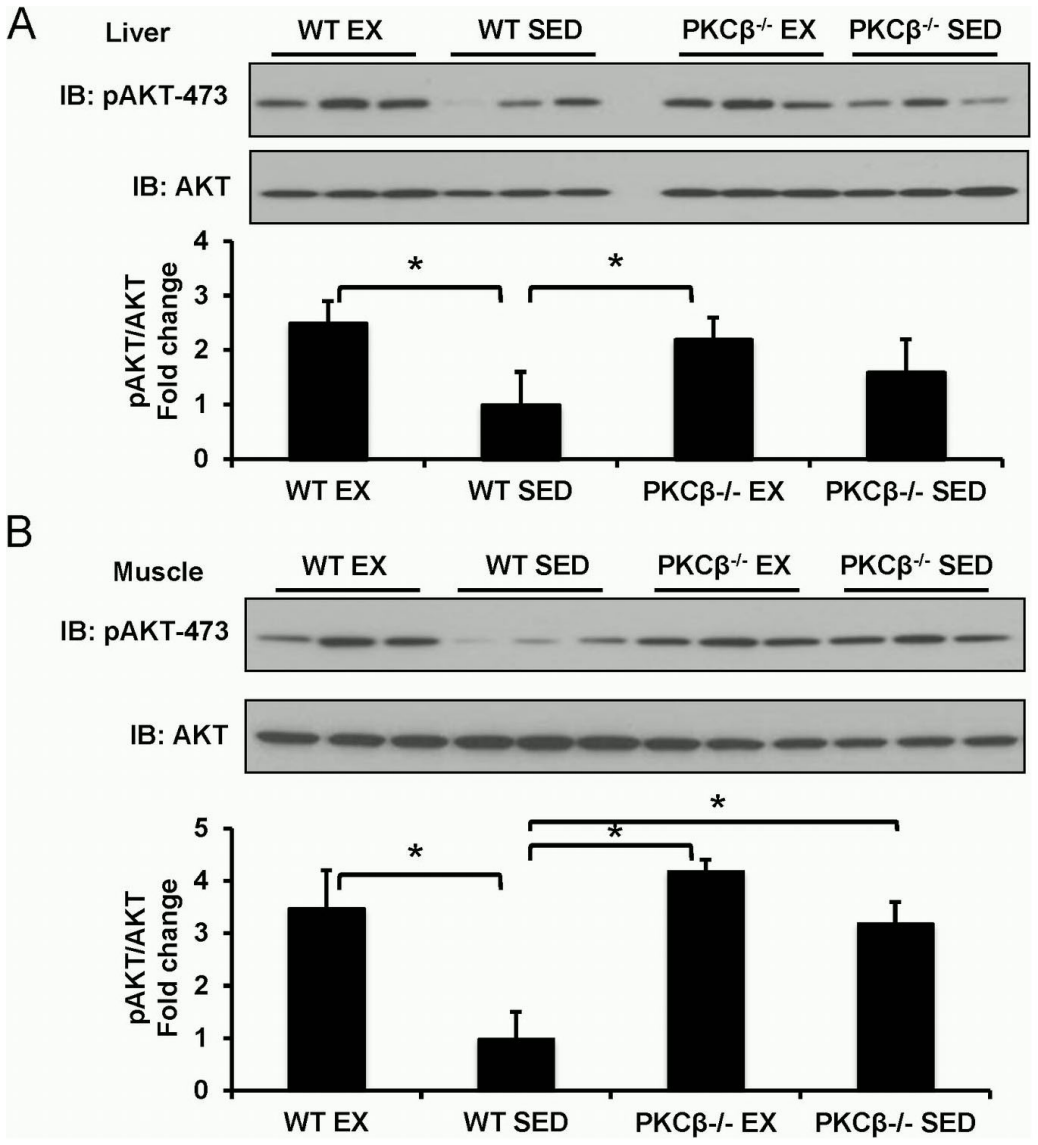
Effect of exercise and PKCβ deficiency on insulin signaling. A, Insulin signal pathway in liver. Insulin downstream signal molecule AKT was activated after exercise in WT mice and had a less effect in PKCβ^-/-^ mice (Top panel, representative images; bottom panel, statistical analysis). *, *P*<0.05. B, Insulin signal pathway in skeletal muscle. Insulin downstream signal molecule AKT was activated after exercise in WT mice and had a less effect in PKCβ^-/-^ mice (Top panel, representative images; bottom panel, statistical analysis). *, *P*<0.05. WT, wild-type; EX, exercise; SED, sedentary; Data are presented as mean ± SEM; n = 3, representative bands from one of 3 independent experiments.

As depicted in Figure 8A, higher activation of AKT was observed in the liver of exercised WT mice, exercised PKCβ^-/-^ mice, and sedentary PKCβ^-/-^ mice when compared with that of sedentary WT mice. Similar results were found in the skeletal muscle (Figure 8B).

## Discussion

Obesity and diabetes are increasing worldwide at an alarming rate largely due to increased prosperity and sedentary life styles. Appropriate diets and exercise are two important interventions for both type 1 and type 2 diabetes. Characterizing the beneficial effects of exercise on insulin sensitivity has been the focus of the research. Despite important advancements in recent years, the mechanistic basis behind how exercise improves insulin signaling is still poorly understood. In the current study, we discovered a potential mechanism by which exercise improved insulin sensitivity in obese subjects.

Expression levels of several isoforms of PKC were altered in skeletal muscle of obese or diabetic patients [5], [6], [7]. Furthermore, forced expression of PKCβ in skeletal muscle caused a decrease in activation of IRS1 and glucose uptake [25]. However, the involvement of PKC proteins in exercise and exercise-mediated metabolic changes is unknown. By detecting different isoforms of PKC, we found that PKCβ levels were significantly decreased in both skeletal muscle and liver, suggesting that PKCβ might be involved in exercise-mediated improvements in insulin sensitivity. Our study further demonstrated that PKCβ deficiency, like exercise, could increase insulin sensitivity, as evidenced by improved ITT and HOMA-IR indices. Similarly, the activation of insulin downstream molecule AKT was enhanced by exercise and PKCβ deficiency. Moreover, no significant differences in insulin sensitivity were observed between exercised and sedentary PKCβ^-/-^ mice, indicating that exercise possibly attenuates insulin resistance via the reduction of PKCβ levels. However, the response of exercised WT mice to glucose challenge in the IPGTT assay was not as significant as that in the ITT, although the blood glucose levels at 0 and 120 min were lower than those of sedentary WT mice. This result is not surprising given the dependence of the IPGTT response on a multitude of factors and this is likely caused by the compensation of insulin secretion, as plasma insulin levels of sedentary WT mice are significantly higher than those of exercised mice.

Consistent with the results reported by other groups [26], [27], exercise lowered HFD-induced weight gain in WT mice. Exercised WT mice and PKCβ^-/-^ mice had a lower fat mass than sedentary WT mice. Of note, no significant effects of exercise on weight gain and visceral fat mass were observed in PKCβ^-/-^ mice. Compared to WT mice, PKCβ^-/-^ mice had lower weight gain in response to HFD feeding, despite comparable food and water intake. This result suggested the PKCβ may affect energy usage. Metabolic measurements indicated that both exercise and PKCβ deficiency enhanced metabolic rate, whereas no effects of exercise on metabolic rate were found in PKCβ^-/-^ mice. Despite less adiposity in PKCβ^-/-^ mice, we did not observe a significant difference in the level of plasma adiponectin between WT and PKCβ^-/-^ mice. This is probably caused by the fact that PKCβ might have a suppressive effect on adiponectin expression. PKCβ has been shown to induce the activation of JNK and subsequently suppress PPARγ, a transcription factor promoting the expression of adiponectin [28], [29].

In addition to insulin sensitivity and metabolism, exercise has beneficial effects on HFD-induced mitochondrial dysfunction, which has been observed in skeletal muscle of both obese rodent and human subjects [30], [31], [32]. Both an HFD and a high-sucrose diet can induce reactive oxygen species production in skeletal muscle, which results in mitochondrial dysfunction [30]. In this study, exercise reduced HFD-induced mitochondrial defects in WT mice. We also observed an ameliorated mitochondrial abnormality induced by HFD (including a more clearly defined internal membrane structure, and appropriate size and number) in both exercised and sedentary PKCβ^-/-^ mice. It has been reported that adipose tissue inflammation contributes to the development of insulin resistance [33], [34]. Recent studies suggest that long-term exercise reduces adipose inflammation via suppression of macrophage infiltration and a switch from M1 to M2 [35], [36]. We detected a slight decrease in macrophage infiltration in exercised WT mice, although no statistical significance was found. We also failed to detect a switch from M1 to M2 in exercised mice, which was probably caused by a shorter exercise period and lower fat content (42%) than studied in previous reports [35], [36]. However, PKCβ deficiency significantly decreased macrophage infiltration in adipose tissue. Considering the fact that an exercise-induced decrease in PKCβ levels in skeletal muscle does not have a significant effect on adipose tissue macrophage infiltration, the decrease in adipose tissue macrophages in PKCβ^-/-^ mice likely resulted from a defect of PKCβ in either adipose tissue or macrophages. In addition, exercise and PKCβ deficiency did not affect the plasma levels of cytokines, including IL-6, IL-10, and MCP-1. These results suggested that 8 weeks of exercise may not have a significant effect on adipose inflammation and that exercise-induced improvements in insulin resistance may be unrelated to changes in adipose tissue macrophage content or function. Circulating level of IL-6 has been reported to be elevated after acute exercise. However, the effect of long-term exercise on IL-6 is controversial [37], [38], [39], [40]. It's reported that no effect of long-term exercise on circulating IL-6 in elder adults, which is consistent with our observations [40], [41].

Taken together, our results suggested that exercise decreased the expression of PKCβ in both skeletal muscle and liver. By reducing PKCβ expression, exercise improved HFD-induced metabolic dysfunction, including insulin resistance, fat accumulation, and mitochondrial dysfunction. Moreover, our findings that PKCβ^-/-^ mice have an increased basal metabolic rate suggest PKCβ could be a potential target for treating obesity and insulin resistance. However, the involvement of entire PKC family in exercise and insulin resistance might be complex due to the diversity of PKC isoforms and potential compensation among different isoforms. It requires further studies to investigate whether the other isoforms of PKC are involved in exercise-mediated improvement of insulin resistance.

## Supporting Information

Figure S1 Exercise regimen. 4-week-old male WT and PKCβ^-/-^ mice were fed a HFD for 16 weeks. At the age of 12 weeks, the mice were randomly assigned into 4 groups: WT exercise (WT EX); WT sedentary (WT SED); PKCβ^-/-^ exercise (PKCβ^-/-^ EX); PKCβ^-/-^ sedentary (PKCβ^-/-^ SED). Mice in EX group were exercise-trained on a motorized treadmill at a speed of 15 m/min, 40 min/day, 5 days/week for 8 weeks. Mice in SED group were put in the treadmill without running 40 min/day, 5 days/week for 8 weeks.
(TIFF)

## References

[1] Hu FB (2011) Globalization of diabetes: the role of diet, lifestyle, and genes. Diabetes Care 34: 1249–1257. 21617109 10.2337/dc11-0442 PMC3114340

[2] Whiting DR, Guariguata L, Weil C, Shaw J (2011) IDF diabetes atlas: global estimates of the prevalence of diabetes for 2011 and 2030. Diabetes Res Clin Pract 94: 311–321. 22079683 10.1016/j.diabres.2011.10.029

[3] Steinberg SF (2008) Structural basis of protein kinase C isoform function. Physiol Rev 88: 1341–1378. 18923184 10.1152/physrev.00034.2007 PMC2899688

[4] Webb BL, Hirst SJ, Giembycz MA (2000) Protein kinase C isoenzymes: a review of their structure, regulation and role in regulating airways smooth muscle tone and mitogenesis. Br J Pharmacol 130: 1433–1452. 10928943 10.1038/sj.bjp.0703452 PMC1572212

[5] Itani SI, Zhou Q, Pories WJ, MacDonald KG, Dohm GL (2000) Involvement of protein kinase C in human skeletal muscle insulin resistance and obesity. Diabetes 49: 1353–1358. 10923637 10.2337/diabetes.49.8.1353

[6] Cooper DR, Watson JE, Dao ML (1993) Decreased expression of protein kinase-C alpha, beta, and epsilon in soleus muscle of Zucker obese (fa/fa) rats. Endocrinology 133: 2241–2247. 8404676 10.1210/endo.133.5.8404676

[7] Itani SI, Pories WJ, Macdonald KG, Dohm GL (2001) Increased protein kinase C theta in skeletal muscle of diabetic patients. Metabolism 50: 553–557. 11319716 10.1053/meta.2001.22512

[8] Geraldes P, King GL (2010) Activation of protein kinase C isoforms and its impact on diabetic complications. Circ Res 106: 1319–1331. 20431074 10.1161/CIRCRESAHA.110.217117 PMC2877591

[9] PKC-DRS Study Group (2005) The effect of ruboxistaurin on visual loss in patients with moderately severe to very severe nonproliferative diabetic retinopathy: initial results of the Protein Kinase C beta Inhibitor Diabetic Retinopathy Study (PKC-DRS) multicenter randomized clinical trial. Diabetes 54: 2188–2197. 15983221 10.2337/diabetes.54.7.2188

[10] Tuttle KR, Bakris GL, Toto RD, McGill JB, Hu K, et al (2005) The effect of ruboxistaurin on nephropathy in type 2 diabetes. Diabetes Care 28: 2686–2690. 16249540 10.2337/diacare.28.11.2686

[11] Huang W, Bansode RR, Bal NC, Mehta M, Mehta KD (2012) Protein kinase Cbeta deficiency attenuates obesity syndrome of ob/ob mice by promoting white adipose tissue remodeling. J Lipid Res 53: 368–378. 22210924 10.1194/jlr.M019687 PMC3276460

[12] Huang W, Bansode R, Mehta M, Mehta KD (2009) Loss of protein kinase Cbeta function protects mice against diet-induced obesity and development of hepatic steatosis and insulin resistance. Hepatology 49: 1525–1536. 19296465 10.1002/hep.22815 PMC2728215

[13] Goldstein MS, Mullick V, Huddlestun B, Levine R (1953) Action of muscular work on transfer of sugars across cell barriers; comparison with action of insulin. Am J Physiol 173: 212–216. 13065430 10.1152/ajplegacy.1953.173.2.212

[14] Devlin JT, Hirshman M, Horton ED, Horton ES (1987) Enhanced peripheral and splanchnic insulin sensitivity in NIDDM men after single bout of exercise. Diabetes 36: 434–439. 3102297 10.2337/diab.36.4.434

[15] Perseghin G, Price TB, Petersen KF, Roden M, Cline GW, et al (1996) Increased glucose transport-phosphorylation and muscle glycogen synthesis after exercise training in insulin-resistant subjects. N Engl J Med 335: 1357–1362. 8857019 10.1056/NEJM199610313351804

[16] Schenk S, Horowitz JF (2007) Acute exercise increases triglyceride synthesis in skeletal muscle and prevents fatty acid-induced insulin resistance. J Clin Invest 117: 1690–1698. 17510709 10.1172/JCI30566 PMC1866251

[17] Goodyear LJ, Kahn BB (1998) Exercise, glucose transport, and insulin sensitivity. Annu Rev Med 49: 235–261. 9509261 10.1146/annurev.med.49.1.235

[18] Leitges M, Schmedt C, Guinamard R, Davoust J, Schaal S, et al (1996) Immunodeficiency in protein kinase cbeta-deficient mice. Science 273: 788–791. 8670417 10.1126/science.273.5276.788

[19] Xu X, Ying Z, Cai M, Xu Z, Li Y, et al (2011) Exercise ameliorates high-fat diet-induced metabolic and vascular dysfunction, and increases adipocyte progenitor cell population in brown adipose tissue. Am J Physiol Regul Integr Comp Physiol 300: R1115–1125. 21368268 10.1152/ajpregu.00806.2010 PMC3094041

[20] Zhong J, Rao X, Deiuliis J, Braunstein Z, Narula V, et al.. (2012) A Potential Role for Dendritic Cell/Macrophage-Expressing DPP4 in Obesity-Induced Visceral Inflammation. Diabetes. 10.2337/db12-0230 PMC3526020 22936179

[21] Xu X, Rao X, Wang TY, Jiang SY, Ying Z, et al (2012) Effect of co-exposure to nickel and particulate matter on insulin resistance and mitochondrial dysfunction in a mouse model. Part Fibre Toxicol 9: 40. 23126276 10.1186/1743-8977-9-40 PMC3545913

[22] Ishizaka N, Ishizaka Y, Toda E, Nagai R, Yamakado M (2005) Association between serum uric acid, metabolic syndrome, and carotid atherosclerosis in Japanese individuals. Arterioscler Thromb Vasc Biol 25: 1038–1044. 15746438 10.1161/01.ATV.0000161274.87407.26

[23] Bal NC, Maurya SK, Sopariwala DH, Sahoo SK, Gupta SC, et al (2012) Sarcolipin is a newly identified regulator of muscle-based thermogenesis in mammals. Nat Med 18: 1575–1579. 22961106 10.1038/nm.2897 PMC3676351

[24] Sun S, Ji Y, Kersten S, Qi L (2012) Mechanisms of inflammatory responses in obese adipose tissue. Annu Rev Nutr 32: 261–286. 22404118 10.1146/annurev-nutr-071811-150623 PMC4041712

[25] Hennige AM, Heni M, Machann J, Staiger H, Sartorius T, et al (2010) Enforced expression of protein kinase C in skeletal muscle causes physical inactivity, fatty liver and insulin resistance in the brain. J Cell Mol Med 14: 903–913. 20569275 10.1111/j.1582-4934.2008.00629.x PMC3823122

[26] Krawczewski Carhuatanta KA, Demuro G, Tschop MH, Pfluger PT, Benoit SC, et al (2011) Voluntary exercise improves high-fat diet-induced leptin resistance independent of adiposity. Endocrinology 152: 2655–2664. 21586558 10.1210/en.2010-1340 PMC3115604

[27] Bhattacharya A, Rahman MM, Sun D, Lawrence R, Mejia W, et al (2005) The combination of dietary conjugated linoleic acid and treadmill exercise lowers gain in body fat mass and enhances lean body mass in high fat-fed male Balb/C mice. J Nutr 135: 1124–1130. 15867292 10.1093/jn/135.5.1124

[28] Liu M, Liu F (2010) Transcriptional and post-translational regulation of adiponectin. Biochem J 425: 41–52. 10.1042/BJ20091045 20001961

[29] He W, Barak Y, Hevener A, Olson P, Liao D, et al (2003) Adipose-specific peroxisome proliferator-activated receptor gamma knockout causes insulin resistance in fat and liver but not in muscle. Proc Natl Acad Sci U S A 100: 15712–15717. 14660788 10.1073/pnas.2536828100 PMC307633

[30] Bonnard C, Durand A, Peyrol S, Chanseaume E, Chauvin MA, et al (2008) Mitochondrial dysfunction results from oxidative stress in the skeletal muscle of diet-induced insulin-resistant mice. J Clin Invest 118: 789–800. 18188455 10.1172/JCI32601 PMC2176186

[31] Kelley DE, He J, Menshikova EV, Ritov VB (2002) Dysfunction of mitochondria in human skeletal muscle in type 2 diabetes. Diabetes 51: 2944–2950. 12351431 10.2337/diabetes.51.10.2944

[32] Kim JA, Wei Y, Sowers JR (2008) Role of mitochondrial dysfunction in insulin resistance. Circ Res 102: 401–414. 18309108 10.1161/CIRCRESAHA.107.165472 PMC2963150

[33] Xu H, Barnes GT, Yang Q, Tan G, Yang D, et al (2003) Chronic inflammation in fat plays a crucial role in the development of obesity-related insulin resistance. J Clin Invest 112: 1821–1830. 14679177 10.1172/JCI19451 PMC296998

[34] Shoelson SE, Lee J, Goldfine AB (2006) Inflammation and insulin resistance. J Clin Invest 116: 1793–1801. 16823477 10.1172/JCI29069 PMC1483173

[35] Bruun JM, Helge JW, Richelsen B, Stallknecht B (2006) Diet and exercise reduce low-grade inflammation and macrophage infiltration in adipose tissue but not in skeletal muscle in severely obese subjects. Am J Physiol Endocrinol Metab 290: E961–967. 16352667 10.1152/ajpendo.00506.2005

[36] Kawanishi N, Yano H, Yokogawa Y, Suzuki K (2010) Exercise training inhibits inflammation in adipose tissue via both suppression of macrophage infiltration and acceleration of phenotypic switching from M1 to M2 macrophages in high-fat-diet-induced obese mice. Exerc Immunol Rev 16: 105–118. 20839495

[37] Oberbach A, Lehmann S, Kirsch K, Krist J, Sonnabend M, et al (2008) Long-term exercise training decreases interleukin-6 (IL-6) serum levels in subjects with impaired glucose tolerance: effect of the -174G/C variant in IL-6 gene. Eur J Endocrinol 159: 129–136. 18469018 10.1530/EJE-08-0220

[38] Nicklas BJ, Hsu FC, Brinkley TJ, Church T, Goodpaster BH, et al (2008) Exercise training and plasma C-reactive protein and interleukin-6 in elderly people. J Am Geriatr Soc 56: 2045–2052. 19016938 10.1111/j.1532-5415.2008.01994.x PMC2683336

[39] Rall LC, Roubenoff R, Cannon JG, Abad LW, Dinarello CA, et al (1996) Effects of progressive resistance training on immune response in aging and chronic inflammation. Med Sci Sports Exerc 28: 1356–1365. 8933485 10.1097/00005768-199611000-00003

[40] Niebauer J, Clark AL, Webb-Peploe KM, Coats AJ (2005) Exercise training in chronic heart failure: effects on pro-inflammatory markers. Eur J Heart Fail 7: 189–193. 15701465 10.1016/j.ejheart.2004.07.012

[41] Moon MK, Cho BJ, Lee YJ, Choi SH, Lim S, et al (2012) The effects of chronic exercise on the inflammatory cytokines interleukin-6 and tumor necrosis factor-alpha are different with age. Appl Physiol Nutr Metab 37: 631–636. 22554223 10.1139/h2012-039

