# Supplementary figures and images for: Exercise Protects against Diet-Induced Insulin Resistance through Downregulation of Protein Kinase Cβ in Mice
Source: PLoS One. 2013 Dec 9;8(12):e81364. doi: 10.1371/journal.pone.0081364 (PMC3857188; doi:10.1371/journal.pone.0081364)

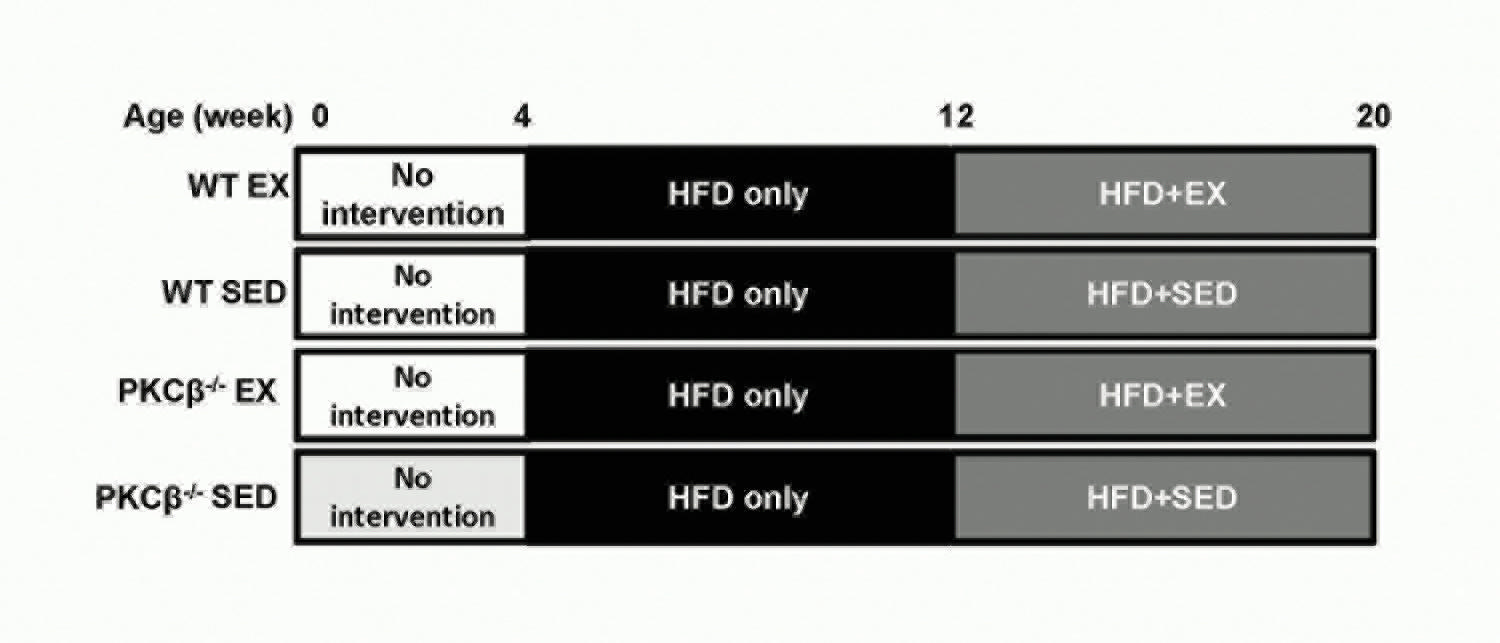

Supplement: Figure S1 — Exercise regimen. 4-week-old male WT and PKCβ-/- mice were fed a HFD for 16 weeks. At the age of 12 weeks, the mice were randomly assigned into 4 groups: WT exercise (WT EX); WT sedentary (WT SED); PKCβ-/- exercise (PKCβ-/- EX); PKCβ-/- sedentary (PKCβ-/- SED). Mice in EX group were exercise-trained on a motorized treadmill at a speed of 15 m/min, 40 min/day, 5 days/week for 8 weeks. Mice in SED group were put in the treadmill without running 40 min/day, 5 days/week for 8 weeks. (TIFF) [file pone.0081364.s001.tiff]
